# Mutations of *γCOP* Gene Disturb *Drosophila melanogaster* Innate Immune Response to *Pseudomonas aeruginosa*

**DOI:** 10.3390/ijms23126499

**Published:** 2022-06-10

**Authors:** Mariana Carmen Chifiriuc, Alexandru Marian Bologa, Attila Cristian Ratiu, Adrian Ionascu, Alexandru Al. Ecovoiu

**Affiliations:** 1 The Research Institute of the University of Bucharest, Faculty of Biology, University of Bucharest, 050095 Bucharest, Romania; carmen.chifiriuc@bio.unibuc.ro; 2 Department of Genetics, Faculty of Biology, University of Bucharest, 060101 Bucharest, Romania; alexandru.bologa@drd.unibuc.ro (A.M.B.); a.ionascu20@s.bio.unibuc.ro (A.I.); alexandru.ecovoiu@bio.unibuc.ro (A.A.E.)

**Keywords:** *γCOP*, *D. melanogaster*, *P. aeruginosa*, innate immunity, Toll, Imd, microarray

## Abstract

*Drosophila melanogaster* (the fruit fly) is a valuable experimental platform for modeling host–pathogen interactions. It is also commonly used to define innate immunity pathways and to understand the mechanisms of both host tolerance to commensal microbiota and response to pathogenic agents. Herein, we investigate how the host response to bacterial infection is mirrored in the expression of genes of Imd and Toll pathways when *D. melanogaster* strains with different *γCOP* genetic backgrounds are infected with *Pseudomonas aeruginosa* ATCC 27853. Using microarray technology, we have interrogated the whole-body transcriptome of infected versus uninfected fruit fly males with three specific genotypes, namely wild-type Oregon, *γCOP^S057302^*/TM6B and *γCOP^14a^*/*γCOP^14a^*. While the expression of genes pertaining to Imd and Toll is not significantly modulated by *P. aeruginosa* infection in Oregon males, many of the components of these cascades are up- or downregulated in both infected and uninfected *γCOP^S057302^*/TM6B and *γCOP^14a^*/*γCOP^14a^* males. Thus, our results suggest that a *γCOP* genetic background modulates the gene expression profiles of Imd and Toll cascades involved in the innate immune response of *D. melanogaster,* inducing the occurrence of immunological dysfunctions in *γCOP* mutants.

## 1. Introduction

*Pseudomonas aeruginosa* is a metabolically versatile opportunistic pathogen, with a very wide host spectrum that includes plants, nematodes, insects, and vertebrates. *P. aeruginosa* can cause a wide range of severe human infections, with high mortality rates, particularly in immuno-depressed patients. A diverse arsenal of *P. aeruginosa* virulence factors, which can have different expression profiles depending on the infection localization and the host immune response, contribute to its high competitiveness [1]. Moreover, *P. aeruginosa* is included in the lists of the most threatening antibiotic resistant pathogens, such as ESCAPE (*Enterococcus faecium*, *Staphylococcus aureus*, *Clostridium difficile, Acinetobacter baumannii*, *Pseudomonas aeruginosa*, and Enterobacteriaceae) and the WHO list of priority one, critical pathogens [2,3]. Besides exhibiting both intrinsic and acquired resistance mechanisms to all currently available antibiotics, *P. aeruginosa* has a high capacity to form biofilms, which are known for their high phenotypic resistance or tolerance to different antimicrobial agents including antibiotics, antiseptics, or host effectors [4].

*P. aeruginosa* infection triggers an intense innate host response in mammals, mainly mediated by neutrophils, resulting in significant host tissue damages. A higher incidence of severe *P. aeruginosa* infections in patients with acquired and primary immunodeficiencies and the better prognoses of the infections with *P. aeruginosa* for patients at the mature age reiterate the importance of the innate immune responses [5,6,7].

Therefore, a better understanding of the interactions among the bacterial and host factors is expected to contribute to the development of improved therapeutic strategies for *P. aeruginosa* infections.

The bacterial infection process and the development of an innate immune system in *Drosophila melanogaster* and mammals have many similarities [8]. An apparent absence of the adaptive immune system and the impressive arsenal of genetic and molecular tools qualify *D. melanogaster* as a powerful experimental platform for investigating the host’s innate immune response [9,10,11,12]. Previous microarray experiments revealed that 283 of the approximately 400 known genes involved in the immune response in *D. melanogaster* are regulated by either one or several signaling pathways [11]. These genes are encoding antimicrobial peptides (AMPs), proteins involved in iron metabolism and sequestration, opsonization, melanization and coagulation processes, production of reactive oxygen species (ROS), signaling via the Jun kinase (JNK) pathway, stress response, and proteases [11]. Toll and Imd cascades are two of the major regulators of the humoral innate immune response in *D. melanogaster* [13]. These pathways are involved in the synthesis and temporary release of AMPs by the cells of the fat body, which is functionally equivalent to the mammalian liver.

In our study, we have harnessed microarray technology in order to investigate the non-lethal effects of experimental infection with *P. aeruginosa* on fruit fly strains with different genetic backgrounds. A goal of this study was to evaluate the impact of normal and disturbed expression of the *γCOP* gene on Toll and Imd pathways consecutive to the infection of males. *γCOP* is an essential, highly conserved gene in eukaryotes and is involved in retrograde vesicular transport between endoplasmic reticulum (ER) and Golgi [14]. Perturbations of the ER functions result in an unbalanced immune response, as ER was linked to numerous processes involved in regulating innate and adaptive immune responses. The main such processes are: the production of inflammatory cytokines, trimming of peptide ligands and assembling of the major histocompatibility complex (MHC) class I molecules, and the transport of both MHC class I and class II complexes to the cell surface [15,16,17,18]. Moreover, *γCOP* is important for the ER homeostasis and hence for the innate immunity, but also for the adaptive immunity as revealed in humans [19].

Our inquiry was relying on a set of particular facts that support a link between the genetic background and the severity of infections. For example, the gut microbiome displays distinct features in complex human diseases and some microbial groups have significant heritability estimates [20]. Noticeably, the survival of mice to *P. aeruginosa* infection is affected by their genetic background, which critically influences the extent of the early cell-mediated innate immunity activation [21,22,23]. Last but not the least, we previously demonstrated that the genetic background of *D. melanogaster* has an impact on commensal microbiota diversity and argued that the natural resistance to the external microbiota colonization could be compromised [24].

## 2. Results

The microarray experiment generated expression data for a total number of 14,437 targets employed by FlyChip procedure based on FL003 arrays. The microarray probes that do not match the target transcripts or match in multiple locations can introduce inconsistency in quantifying gene expression [25]. Therefore, we used for data analysis only single hit/matched genes assigned to the oligonucleotide sequence. Consecutively, we focused on Imd, Toll, and Imd-JNK pathways and considered for further analyses 99 specific genes and *γCOP*.

**Figure 1:**
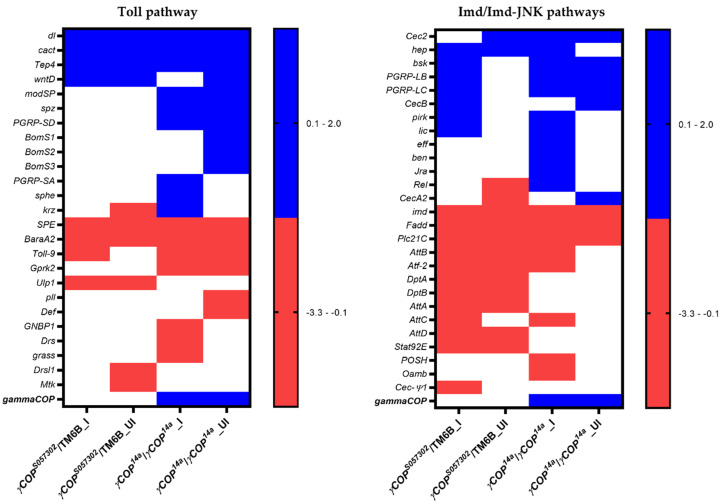
Heatmaps of genes having a significant expression variation in at least one mutant background. The gene names are indicated on the left side of each heatmap (Toll pathway and Imd/Imd-JNK pathways). For each infected (I) or uninfected (UI) experimental condition, downregulated genes are colored in red, while the upregulated genes are indicated with blue. The white squares indicate that the expression variation was not statistically significant. The gene expression is expressed as log_2_FC.

An overview of the 51 genes significantly (*p* < 0.05) modulated in at least one category of uninfected and infected *γCOP^S057302^*/TM6B or *γCOP^14a^*/*γCOP^14a^* mutant males (Table 1 and Figure 1) suggests that a *γCOP* genetic background influences the innate immune response, even in the absence of infection. We also considered for further analyses the gene expression of *Drsl1* in uninfected *γCOP^S057302^*/TM6B, for which we found a P value of 0.059, bringing the total number of represented genes to 52. Regarding *AttB* and *CecA2*, we deemed as significant their gene expression in uninfected *γCOP^S057302^*/TM6B and, respectively, uninfected *γCOP^14a^*/*γCOP^14a^* mutant males (*p* = 0.059 and *p* = 0.056).

As revealed in Figure 1, *γCOP* expression is not affected in either infected or uninfected heterozygous mutant males. Nevertheless, it deserves mentioning that *γCOP* have a tendency to downregulation in both categories of *γCOP^S057302^*/TM6B males as revealed by the fact that all eight log_2_FC values standing for the 4 × 2 biological replicates are negative.

The biological processes indicated in Table 1 are retrieved from FlyBase [26] and are in accordance to the FlyBase release FB2022_02.

Hence, we obtained four sets of differentially expressed genes for the four categories of mutant males, namely 27 genes for infected *γCOP^S057302^*/TM6B, 24 genes for uninfected *γCOP^S057302^*/TM6B, 35 genes for the infected *γCOP^14a^*/*γCOP^14a^*, and 25 genes for uninfected *γCOP^14a^*/*γCOP^14a^*. A summation of the gene expression profiles in the considered experimental conditions is presented in Figure 1. Strikingly, none of the selected genes is significantly up- or downregulated in the infected Oregon males.

No less than 10 genes from the Toll pathway have an affected expression pattern in both infected and uninfected *γCOP^14a^/γCOP^14a^* males. In fact, out of these genes, six are upregulated (*dl*, *cact*, *Tep4*, *modSP*, *spz* and *PGRP*-*SD*) and four are downregulated (*SPE*, *BaraA2*, *Toll-9,* and *Gprk2*). Both categories of *γCOP^S057302^*/TM6B males share only seven modulated genes, as *dl*, *cact*, *Tep4,* and *wntD* are upregulated, while *SPE*, *BaraA2*, and *Ulp1* are downregulated. On the other hand, three upregulated genes (*dl*, *cact,* and *Tep4*) and two downregulated genes (*SPE* and *BaraA2*) are common for both homozygous and heterozygous mutant males.

A similar analysis of Imd/Imd-JNK pathways revealed that, out of 27 considered genes, only 3 of them are mutual (*imd*, *Fadd,* and *Plc21C*) and are downregulated in all four categories of mutant males. When considering the *γCOP^14a^/γCOP^14a^* infected and uninfected males, in a group of seven shared genes we found four of them to be upregulated (*Cec*2, *bsk*, *PGRP-LB,* and *PGRP-LC*) and three downregulated (*imd*, *Fadd,* and *Plc21C*). On the other hand, there are 11 genes displaying a similar expression pattern in both categories of *γCOP^S057302^*/TM6B males, where 1 gene is upregulated (*hep*) and 10 genes are downregulated (*imd*, *Fadd*, *Plc21C*, *AttB*, *Atf*-*2*, *DptA*, *DptB*, *AttA*, *AttD,* and *Stat92E*).

In the Toll pathway, the number of genes with impacted expression is bigger for the default (uninfected) condition in *γCOP^14a^/γCOP^14a^* compared to *γCOP^S057302^*/TM6B males (16 genes versus only 10 genes), suggesting that the *γCOP^14a^/γCOP^14a^* males set up an enhanced response to a commensal microbiota challenge. When scrutinizing inside of the same genetic background, there are only very small numeric differences (Δ) of the affected genes in infected versus uninfected flies (for *γCOP^14a^/γCOP^14a^* males Δ = 0 and for *γCOP^S057302^*/TM6B males Δ = −2). Remarkably, six of the genes modulated in the infected *γCOP^S057302^*/TM6B males are also affected in the infected *γCOP^14a^/γCOP^14a^* flies. Only *wntD* and *Ulp1* genes are specifically responsive to infection with *P. aeruginosa* in heterozygous flies.

Concerning the Imd/Imd-JNK pathways, uninfected *γCOP^14a^/γCOP^14a^* males seem to be less reactive, as long as they exhibit by default only 9 affected genes comparative to 14 genes modulated in the uninfected *γCOP^S057302^*/TM6B flies (Δ = −5). Conversely, there is a double increment in the number of the affected genes in the infected *γCOP^14a^/γCOP^14a^* mutant males (Δ = 10; from 9 to 19 genes) comparative to the infection response of *γCOP^S057302^*/TM6B mutant males (Δ = 5; from 14 to 19 genes). Coincidentally, 19 genes (Δ = 0) from Imd/Imd-JNK pathways are modulated in both infected *γCOP^14a^/γCOP^14a^* and infected *γCOP^S057302^*/TM6B males. Out of these 19 affected genes, the two categories of infected males share six upregulated genes (*hep*, *bsk*, *PGRP-LB*, *PGRP-LC*, *pirk* and *lic*) and six downregulated genes (*imd*, *Fadd*, *Plc21C*, *AttB*, *Atf2* and *AttC*). It is somehow difficult to avoid noticing the symmetry of this numbers.

**Figure 2:**
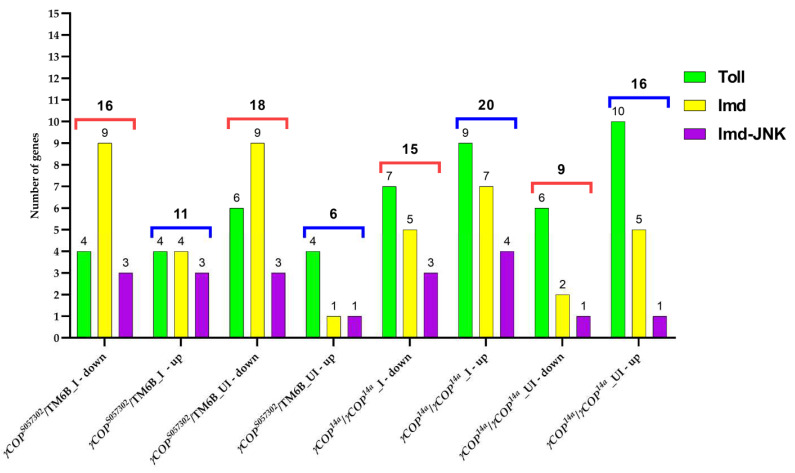
An overview of the differential expression of immune genes in *γCOP* mutant males. For every infected (I) or uninfected (UI) experimental condition, we counted the number of genes pertaining to Imd, Toll, and Imd-JNK pathways that were significantly down- or upregulated. Red (for downregulated genes) and blue (for upregulated genes) lines are highlighting the differences between the I/UI heterozygous and homozygous males.

In the absence of infection, the Toll pathway appears to be more active in *γCOP^14a^/γCOP^14a^* mutants comparative to the heterozygous males, but for both considered genetic pathways, the experimental infection impacts more on gene modulation in the *γCOP^14a^/γCOP^14a^* mutant males. However, the upregulation of some genes encoding for pathway inhibitors invite careful interpretation of these aggregate data (Figure 2).

## 3. Discussion

### 3.1. An Overview of the Mutant γCOP Background in D. melanogaster

Herein, we investigate if the essential gene *γCOP* is involved in modulation of the innate immune response in *D. melanogaster*. In order to test our hypothesis, we performed experimental infection of males from Oregon, *γCOP^S057302^*/TM6B, and *γCOP^14a^/γCOP^14a^* lines with the *P. aeruginosa* ATCC 27853 strain.

In our experiments, we used the fruit fly strain *γCOP^S057302^*/TM6B, which contains the null lethal allele *γCOP^S057302^* balanced over the TM6B chromosome. Therefore, the *γCOP^S057302^* allele is unable to migrate in the TM6B balancer and is maintained in its location on the original *l(3)S057302* chromosome [27]. The *l(3)S057302* chromosome is a specific third chromosome of *D. melanogaster* obtained by insertional mutagenesis [27] and harbors an insertion of transposon *P{lacW}^S^*^057302^ in the 5′UTR region of the *γCOP* gene [28]. The insertion determines a recessive lethal allele as revealed by obtaining precise viable excisions of *P{lacW}^S^*^057302^—proof that there is no other hidden mutation in the *l(3)S057302* chromosome accountable for the lethal phenotype [28]. Moreover, we found that *γCOP^S057302^* fails to complement the lethality of *γCOP^∆114^* (located in a chromosome 3 with a different mutational background than *l(3)S057302*), confirming data of Jayaram et al. [29] and the fact that *γCOP* is indeed an essential gene, being also involved in fruit flies’ fertility [30]. The crosses among *γCOP^14a^/γCOP^14a^* males and females are always sterile, but both homozygous males and females are fertile when crossed with flies having at least one wild-type (WT) copy of *γCOP* (*γCOP^WT^*).

TM6B balancer contains multiple inversions and rearrangements, but also recessive lethal genetic markers causing the homozygous individuals to die as embryos. These chromosomal aberrations prevent recombination by meiotic crossing over with the normal homologous chromosome 3. Herein, the term “normality” refers only to the natural linkage order of the genes, but not to alleles and or to other small-scale mutations. A balanced chromosome is actually a haploblock, which keeps in cis configuration not only the lethal recessive alleles, but also other known or hidden mutations, considering that the balancer is not leaky [31]. TM6B contains the spontaneous inversion *In(3R)C,* which is also present in many natural populations but not in the Oregon strain [26]. The breakpoints of *In(3R)C* are 92D and 100F; *γCOP* is located in 100C, close to the distal breakpoint of this inversion. Detailed data concerning the multiple inversions’ effects on the expression of genes located in fruit fly balancer chromosomes were reported [32]. Misexpression of genes is determined either by their proximity to breakpoints of inversion or because they are physically disrupted by the inversions [31].

As revealed by Bing et al. [33], the impact of transvection on gene regulation is a complex and a plastic phenomenon. Transvection (genetic trans-interaction) as a particular way of gene regulation is widespread on the *D. melanogaster* genome. The same authors prove that trans-interactions at the *Malic enzyme* (*Men*) locus were eliminated by inversions of two different chromosomal fragments that contain *Men* [33]. The authors report that the sole wild-type *Men* allele is upregulated by the enhancers of a null *Men* allele, and the relative quantity of the specific mRNA and enzyme recovers to those of homozygous wild-type organisms. Bing et al. [33] further proved that *Men* upregulation of the wild-type was indeed a transvection, as inversions containing *Men* wild-type locus prevents its compensatory upregulation. The temperature was also an impeding factor, pointing to an environmental influence on the plasticity of transvection aspects.

Since the *γCOP^S057302^*/TM6B strain contains a null allele and a relocated copy of *γCOP^WT^*, impairments of the anterograde and retrograde transport between Golgi and ER may be reasonably considered if the *γCOP^WT^* allele’s physiology is disturbed by position effects or by defective trans-interactions. On the other hand, *γCOP^14a^/γCOP^14a^* males are homozygous for two hypomorphic alleles [30]; hence, neither of the two mutant strains contains a typical WT copy of the *γCOP* gene comparative to the Oregon control flies.

We suppose that alternative compensatory mechanisms are acting in the mutant background of the two strains. The overexpression of *γCOP* in *γCOP^14a^*/*γCOP^14a^* males does not necessarily result in an overproduction of γCOP protein since the *γCOP^14a^* allele proved to be functionally hypomorphic. The qRT-PCR and microarray data show a compensative overexpression of an abnormal mRNA encoded by the *γCOP^14a^* allele, actually conducting a net deficit of the protein comparative to Oregon males [30]. On the other hand, *γCOP^S057302^* is an embryo lethal null allele, so an upregulation is not expected for it, but it is for the wild-copy allele, by a transvection phenomenon similar to the one reported for the *Men* gene [34]. Hence, it is plausible to consider that in the heterozygous males, In(3R)2C impedes on the ability of the specific cis-regulating sequences to enhance the expression of *γCOP* by trans-regulation of the normal allele. Alternatively, this inversion may also expose *γCOP* to the action of local non-specific suppressors, resulting in a slight downregulation of gene expression [35].

In a previous work focusing on a commensal microbiota load of our mutant strains [24], we found that *γCOP^14a^*/*γCOP^14a^* males harbored the lowest bacterial load as compared to both *γCOP^14a^*/TM6B and Oregon flies, the latter having the highest microbial charge [24]. Lowering the rearing temperature to 18 °C amplifies the bacterial load differences between *γCOP^14a^/γCOP^14a^* and Oregon individuals. These differences may be a consequence of impaired intracellular vesicle cargo protein transport that could impact protein secretion and the assembly of receptors involved in microbial recognition.

The data fit the same hierarchy of the allelic severity scale that we previously reported for lethality and sterility of these *γCop* alleles [30]. Therefore, the phenotypic severity modulation of lethality, sterility, and commensal microbiota load are reasonably accountable for the differences between the two alleles of *γCOP*. If commensal load data was inconsistent with the severity of *γCOP* alleles, we would have suspected a hidden mutation in *l(3)S057302* chromosome.

A recent paper [19] reports that homozygous missense mutation p.K652E in human γ1-COP impedes on the ability of COPI complex to bind to the KDEL receptor (KDELR), a key step in the retrieval of chaperones containing the KDEL domain from Golgi to endoplasmic reticulum (ER). The KDEL chaperone proteins are required in ER to assist the proper folding of the secretory proteins as antibodies and cytokines, and their absence contributes to ER stress. Both human patients and model homozygous *Copg1^K652E^* mice are affected by combined immunodeficiency (CID), defined by disturbed humoral and cell-mediated immunity. Therefore, the authors demonstrate that p.K652E in the homozygous condition is responsible for CID and reveal that the *COPG1* gene is required for a normal adaptive immune response.

In this study, we have shown that, upon *P. aeruginosa* ingestion, the *γCOP* heterozygous or homozygous mutants exhibit a different expression profile of many genes encoding for highly evolutionarily conserved innate immune receptors, signaling pathways, and effector molecules, which might affect the response to both pathogenic agents and normal microbiota. When focusing on microarray results for genes from Toll and Imd pathways involved in the innate immunity, we noticed intriguing results. Significant gene expression modulations in either infected or uninfected flies are more frequent in *γCOP^14a^/γCOP^14a^* males as compared to *γCOP^S057302^*/TM6B ones, and are more frequent in Toll rather than in Imd pathways (Figure 1 and Figure 2).

Our microarray data reveal that the transcription patterns of Toll and Imd/Imd-JNK pathways in *γCOP^14a^/γCOP^14a^* males are associated with a significant upregulation of *γCOP* (3.8079X for the infected males and 2.4478X for the uninfected ones). We previously detected a comparative level of overexpression in testes and the oocytes of *γCOP^14a^/γCOP^14a^* individuals, but also in *γCOP^14a^/γCOP^14a^* embryos [30]. These constitutive, correlated expression profiles support once more that *γCOP* mutant background is, at least partially, responsible for the genes’ expression snapshot noticed in Toll and Imd/Imd-JNK pathways.

The genetic background of the organisms used in biology experiments are always a sensible aspect of data interpretation. Although we rely on complementation tests for assessing the *γCOP* alleles, hidden or known mutations are to be considered when making conclusions about the results. Sometimes, more or less gratuitous genetic markers are employed in crosses of fruit flies in order to ease the trace back of mutant alleles of the genes of interest. In our case, both *γCOP^14a^*/*γCOP^14a^* and *γCOP^S057302^*/TM6B males have constitutively hemizygous mutations for *white*, a gene located in the X chromosome and recently reported to be involved not only in red eye pigmentation, but also in the response to some metabolic and cellular stresses [36,37]. To date, there is no information indexed in FlyBase to support or suggest that *white* is involved either in the immune response of flies or in interactions with *γCOP* or COPI. Nevertheless, in order to exclude a potential contribution of *white* to the activation of Toll and Imd/Imd−JNK pathways in our experiments, we took advantage to the fact that *γCOP^S057302^*/TM6B males contain a single functional copy of *mini-white* (*w^+mC^*) allele in the *P{lacW}^S^*^057302^ transposon [26]. The *w^+mC^* is a key genetic marker used to confirm a *P{lacW}* insertion in the genome of fruit flies with *white* defective genetic background. Since all of the *γCOP^S057302^*/TM6B males have deep orange eyes instead of white ones, it is evident that *w^+mC^* is functional in these transgenic organisms. Our microarray data reveal that the expression level of *white* in the uninfected and infected heterozygous males is indeed the same as in the Oregon ones, which contain a wild-type copy of *white*. The *γCOP^14a^* allele was obtained by inducing an imprecise excision of *P{lacW}^S^*^057302^, therefore *γCOP^14a^*/*γCOP^14a^* males no longer contain the *w^+mC^* allele. As expected, the microarray data show a significant similar downregulation of *white* expression in both uninfected and infected *γCOP^14a^*/*γCOP^14a^*, but the behavior of Toll and Imd/Imd−JNK are still similar to the *w^+mC^*, *γCOP^S057302^*/TM6B males. Altogether, these genetic and molecular data reasonably rule out a significant contribution of the *white* background in the activation of Toll and Imd pathways in the *γCOP^S057302^*/TM6B males, or in keeping these pathways in a dormant state in the wild-type *white* background of Oregon flies. Although over a thousand specific alleles are reported in FlyBase, *white* is not an essential gene, as no lethal alleles of it are documented so far. Conversely, excepting for *εCOP*, lethal alleles are reported for 7 out of 8 genes encoding for COPI complex in *D. melanogaster*.

It is remarkable that none of the genes scrutinized in this paper are either up- or downregulated in infected Oregon males. Plausibly, the *γCOP* background is the genetic driver that modulates the expression of many Toll and Imd genes in the uninfected mutant homozygous and heterozygous males, but not in the Oregon ones. Our findings are consistent with the essential role of γCOP proteins in the assembly of COPI vesicles; γCOP is the main interactor of the ADP-ribosylation factor at 79F (Arf79F), the fruit fly ortholog of mammalian ARF-1, which, in its turn, is a key protein for both assembly of the COPI complex and for anchoring it to the membrane of Golgi. The other interactor of Arf79F is βCOP (encoded by *βCOP* in *D. melanogaster*) and disruption by RNAi of either gene influence the immune system response of *D. melanogaster* [38,39]. A very conspicuous aspect is that the depletion of Arf79F by RNAi activates the Toll pathway in fruit flies, contributing to the overexpression of *Drs* and *Mtk* [38] even in the absence of an experimental infection with Gram-positive bacteria. These data partially mirror our data for uninfected *γCOP^S057302^*/TM6B males, where *Mtk* and *Drsl1* are constitutively downregulated, but *dl*, *cact*, *Tep4,* and *wntD* are upregulated.

*Arf79F*, *βCOP,* and *γCOP* represent the key triplet involved in COPI assembly. Since *Arf79F* and *βCOP* are associated with the immune system GO term in FlyBase, it is logical to learn that *γCOP* is also involved in the immune response of *D. melanogaster*.

Our experiments revealed that Toll and Imd/Imd-JNK pathways are not activated in the infected Oregon males mainly because they are wild-type for *γCOP*. Oregon males respond to *per os* infection with *P. aeruginosa* but without involving genes of Toll and Imd pathways. The Oregon males exhibit an immune response to these bacteria, as *ventral veins lacking*, *Listericin*, *Turandot A* and *Turandot C* genes are modulated consecutively with infection. On the other hand, experimentally infected Oregon strain may not activate some genes involved in the innate immune response, most probably if a distinct infection is interpreted as a relatively mild one. As an example, a *per os* infection with the flagellate parasite *Crithidia* spp. activates *CecA1* and *Diptericin* genes from the Imd pathway and *Def* and *Drs* from the Toll pathway only in the ILL97 wild-type strain but not in the Oregon [40] flies.

The biological significance of the mutant background of *γCOP* (at least in males) is that disturbances in the normal physiology of *γCOP* impact Toll and Imd/Imd-JNK pathways and induce modulations of the transcriptional profiles for many of the constituent genes. Albeit the homozygous mutants present a robust overexpression and the heterozygous display a questionable downregulation of *γCOP*, it appears that both conditions have an impact on the pathways. Since misregulation of *γCOP^14a^* is stronger and significant, it is expected to have a greater impact on the transcriptional landscape of both pathways. Overexpression of *γCOP^14a^* in *γCOP^14a^*/*γCOP^14a^* males determines a more consistent perturbation of the Toll pathway and a bigger difference between infected versus uninfected homozygous males.

We inquire further about the genes from the Toll and Imd/Imd-JNK pathways that had a significantly modulated gene expression in our experiment.

### 3.2. Toll Pathway

In contrast to most vertebrate species, the *D. melanogaster* anti−infectious defense is represented by highly conserved, complex, and interrelated innate immunity mechanisms, which are activated by the recognition of pathogenic viruses, bacteria, fungi, and parasites [41]. Regarding *P. aeruginosa*, previous studies have shown that besides the well-known pro-inflammatory effects of LPS, many other virulence factors, including proteins involved in protein folding, quorum sensing, transcriptional regulators, efflux (multidrug transporters) systems, biosynthesis of redox active compounds (periplasmic thiol-disulfide oxidoreductase), toxins (exotoxin A, phospholipase C, repeats-in-toxin exoproteins), and proteins of unknown function are essential for maximum pathogenicity in *D. melanogaster*, triggering the activation of an innate immune response and secretion of proinflammatory cytokines [42,43,44].

Toll and Imd are the major innate immunity pathways regulating the production of AMPs and other immune responses in fruit flies [45].

**Figure 3:**
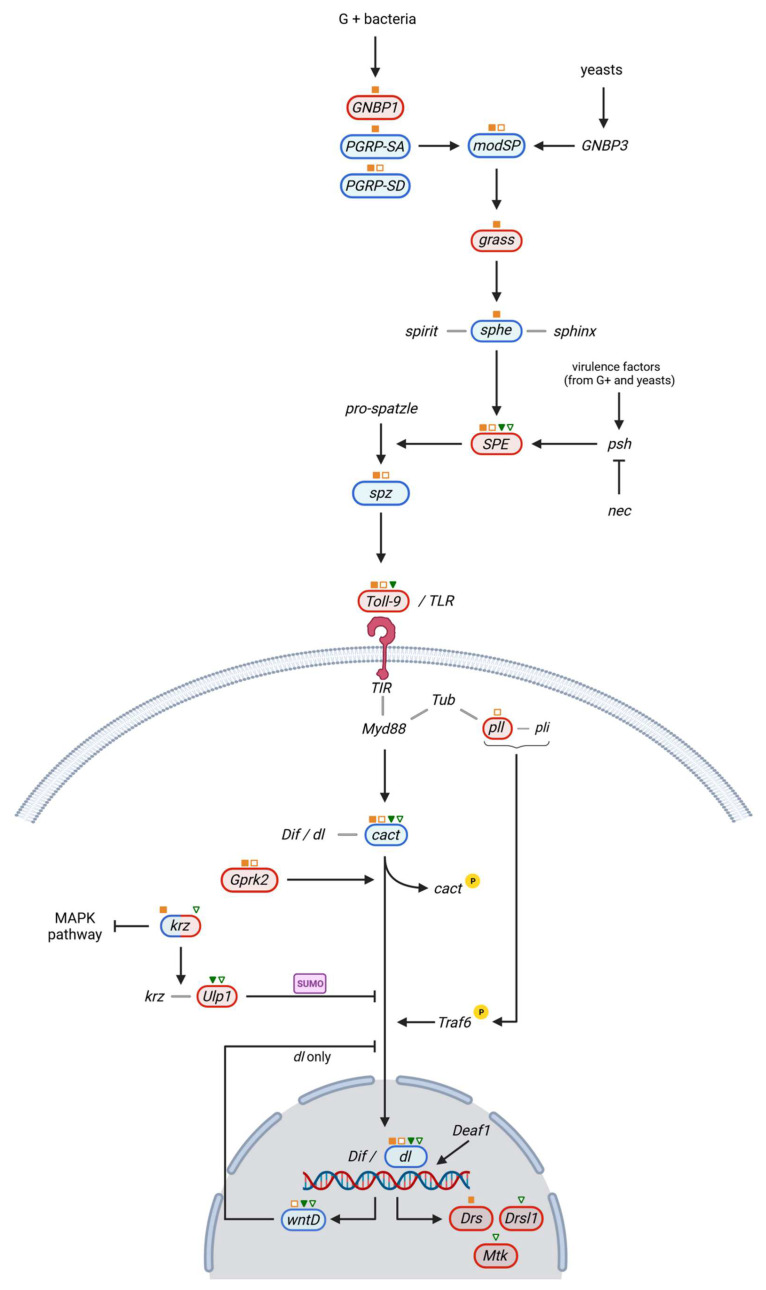
Biochemical model of Toll pathway activation in *D. melanogaster* cells as a response to microbial infection. The observed gene expression change corresponding to the experimental conditions and mutants is shown in blue (upregulation) or red (downregulation). With orange-filled or orange-bordered squares, we indicate that the corresponding gene significantly varies its expression in infected and, respectively, uninfected *γCOP^14a^/γCOP^14a^* males. With green-filled or green-bordered triangles we indicate that the marked gene significantly varies its expression in infected and, respectively, uninfected *γCOP^S057302^*/TM6B males. Unmarked components of the pathway did not show any statistically significant expression change. Created with BioRender.com (accessed on 24 February 2022).

The Toll/Cactus signaling pathway is mainly triggered by fungal infections, while the Imd cascade, including TAK1, Dredd, IKK complex, and Rel, is activated in bacterial infections [46,47]. The Toll pathway (Figure 3) is activated upon the binding of spz cytokine to the Toll-1 receptor ectodomain. Upon receptor signaling, dimerization/oligomerization of TIR (Toll/IL-1R receptor), the intracellular domain of the Toll receptor, allows the recruitment of a complex comprising of Myd88, Tub and pll. This complex is responsible for downstream phosphorylation and degradation of cact, facilitating the nuclear translocation of dl and dorsal-related immunity factors (Dif) [48]. Cact phosphorylation is catalyzed by Gprk2 [49]. Dif/dl nuclear translocation is positively regulated by TNF receptor-associated factor 6 (Traf 6), following interaction with the pll and Pellino (pli) complex [50]. This process is also negatively regulated by a negative feedback loop involving wntD [51] and a krz-Ulp1 sumorylation complex [52]. Dif/dl dimers or heterodimers require a deformed epidermal autoregulatory factor-1 (Deaf1) transcription factor to produce the antimicrobial peptides Drs, Drsl1 and Mtk [48].

Eight other Toll receptors (Toll 2–9) are described in *Drosophila,* although their involvement in the upregulation of AMP genes is not fully understood. However, regarding Toll-9, it was demonstrated that vertebrates express multiple Toll-like receptors (TLRs) resembling *Drosophila* Toll-1 receptor. They are involved in the regulation of different immune responses upon recognition of different pathogen-associated molecular structures, such as Gram-negative bacterial lipopolysaccharides, peptidoglycans, or flagellin [48].

Pathogen recognition is dependent on the interaction between the extracellular modSP and GNBP1, GNBP3 or PGRP-SA, and PGRP-SD [53], leading to an extracellular cascade involving grass and a serine protease complex (spirit, spheroid, sphinx) resulting in the activation of SPE, responsible for spz activation [48]. In our study, the expression of *SPE* was downregulated in all four mutant lines, irrespective to the infection, demonstrating that the genetic background inhibits the production of spz cytokine, the ligand of Toll-9. Moreover, the constitutive expression of Toll-9 is inhibited in the infected and uninfected homozygous mutants, as well as in the infected heterozygotes, proving the role of the genetic background in the innate immune response. Previously, it was demonstrated that Toll-9 is functionally homologous to Toll-1 inducing a strong *dl* expression [54].

Regarding the Toll cascade inside the nucleus, we have observed that in both infected and uninfected experimental conditions, *cact* is upregulated. To bind to Toll-1, the circulating zymogen (pro-spz-1) is cleaved by the SPE into spz [55]. Subsequently to spz binding to Toll-1, the I-κB inhibitor cact is phosphorylated and degraded, enabling the translocation of the NF-κB transcription factors Dif and dl into the nucleus where they stimulate the AMP genes expression [56]. Thus, one would expect that the upregulation of *cact* could block the nuclear translocation of the dl transcription factor. However, in our study the *dl* expression was increased in all experimental groups.

The *γCOP* mutant background is also associated with increased expression of the *wntD*. It was previously shown that wntD acts as a feedback inhibitor of dl/NF-κB, blocking dl nuclear accumulation, independently of the I-κB homologue cact. The *wntD* loss-of-function (LOF) was associated with immune defects and increased levels of Toll/dl signaling [57]. However, in our study both *wntD* and *dl* expression levels were increased. Thus, our results suggest that in *γCOP* mutants, *dl* expression levels are increased, even though two of the inhibitors of this pathway, i.e., *cact* and *wntD*, are upregulated. This might suggest the involvement of other regulatory pathways.

The downregulation of the SUMO protease, *Ulp1*, correlated with decreased expression of the β-arrestin *krz,* was noticed in the heterozygous *γCOP* mutants. The *Ulp1* downregulation could also account for the dysregulation of the Toll signaling pathway and impairment of the immune system homeostasis. Previous studies have shown that *Ulp1* or *krz* LOF were accompanied by an increased inflammatory response in *Drosophila* larvae [52].

The Toll pathway seems to be more severely affected in homozygous *γCOP* mutants via the modulation of the events upstream of the Toll receptor, i.e., downregulation of *GNBP1* and upregulation of *PGRP*-*SA*, *PGRP*-*SD,* and *ModSP*. ModSP is a modular serine protease that was shown to interconnect the circulating recognition molecules with the grass-SPE-spz extracellular pathway upstream of the Toll receptor [53]. Going downstream of the Toll receptor, the innate response of the homozygous *γCOP* mutants is additionally impaired by the downregulation of the highly conserved *pll* (coding for a serine/threonine protein kinase), a positive regulator of the nuclear translocation of the NF-κB transcription factors Dif and dl [58]. Interestingly, *Gprk2* was inhibited only in homozygous mutants. This kinase has an evolutionarily conserved role in normal *dl* expression through a NF-κB regulation mechanism, which is unrelated to cact degradation. Noticeably, the cytokine expression consecutive to *Escherichia coli* infection was impaired in zebrafish embryos for the orthologous GRK5, and in *D. melanogaster*, the susceptibility of infections with *Enterococcus faecalis* infection is increased by RNAi silencing of *Grpk2* [49].

Our results demonstrate for the first time that mutations in *γCOP* alter the proper function of this conserved NF-κB signaling regulation pathway. Consequently, the fruit fly’s capability to respond adequately to Gram-negative bacterial infections is also perturbed.

Overall, our data suggest that the *γCOP* background interferes with the Toll signaling pathway and with the innate immunity of *Drosophila*. Most probably, this interaction affects the fruit fly’s ability to recognize the pathogen-associated molecular patterns and to further activate the signal transduction cascades and the transcription of effector genes.

### 3.3. Imd Pathway

The second major cascade involved in the host–pathogen crosstalk is the Imd pathway, whose main role is the production of AMPs through the NF-κB transactivator Rel.

**Figure 4:**
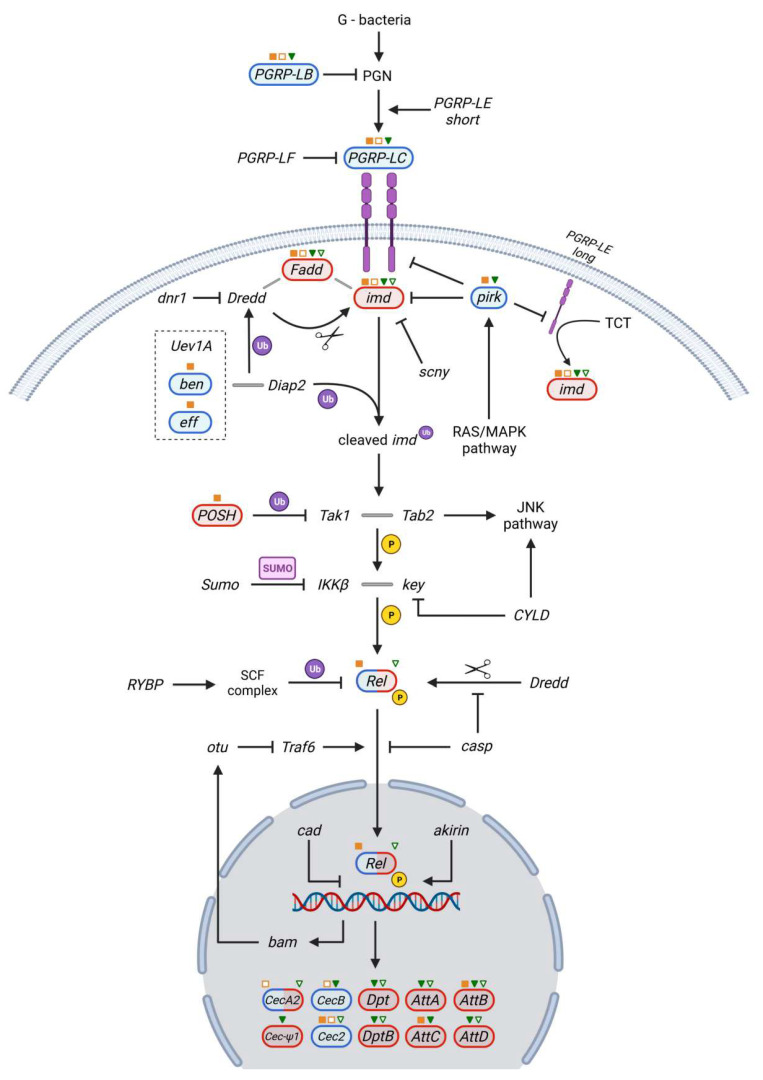
Biochemical model of Imd pathway activation in *D. melanogaster* cells as a response to Gram-negative (G-) infection. The observed gene expression change corresponding to the experiment conditions and mutants is shown in blue (upregulation) or red (downregulation). With orange-filled or orange-bordered squares we indicate that the pinpointed gene significantly varies its expression in infected and, respectively, uninfected *γCOP^14a^/γCOP^14a^* males. With green-filled or green-bordered triangles we indicate the genes that significantly vary their expression in infected and, respectively, uninfected *γCOP^S057302^*/TM6B males. Unmarked components of the pathway did not show any statistically significant expression change. Created with BioRender.com (accessed on 24 February 2022).

The Imd pathway (Figure 4) shares many features with the mammalian TLR/IL-1 and TNF-R signaling pathways, pointing to a highly evolutionary conserved innate immunity mediated by production of AMPs [59,60].

Imd pathway-mediated pathogen recognition is dependent on the interaction between LPS and other Gram-negative bacterial wall components, such as the *meso*-diaminopimelic acid (DAP)-type peptidoglycan (PGN) and the PGRP-LC transmembrane receptor [61,62]. Additional PGRP proteins are involved in PGN regulation (PGRP-LB) and receptor binding (PGRP-LE and PGRP-LF) [63]. The intracellular membrane bound receptor PGRP-LE plays a crucial part in the immune response against intracellular pathogens by cooperatively regulating expression of intracellular antimicrobial peptide Listericin via a JAK/STAT interaction [64].

Upon receptor activation, an intracellular complex is recruited at the receptor site, consisting of imd, Fadd, and Dredd (caspase-8 homolog Death-related ced-3/Nedd2-like protein). Ubiquitination of Dredd by Diap2, Uev1A, ben, and eff complex activates Dredd for the cleavage of an imd N-terminal site, followed by ubiquitination of cleaved imd [65]. Alternatively, activation of the intracellular membrane bound receptor PGRP-LE by tracheal cytotoxins (TCT) results in similar interactions. At this level, pirk, an imd and RAS/MAPK pathway regulated protein, inhibits imd activation by the transmembrane receptors [66]. Processed imd activates downstream the Tak1, Tab2 complex, which phosphorylates the IKK complex (IKKβ and key), leading to activation of Rel transcription factor [63]. Prior to nuclear translocation, phosphorylated Rel requires Dredd mediated cleavage. Whilst Caspar (casp) inhibits both the Dredd-Rel interaction and Rel nuclear translocation, Traf6 facilitates the latter [63,67]. Transcription factors caudal (cad) [68] and nuclear protein akirin [69] interact with Rel dimers for regulating the production of several types of AMPs, namely diptericins (Dpt, DptB), cecropins (CecA2, CecB, Cec-Ψ1, Cec2) and attacins (AttA, AttB, AttC, AttD).

In our study, both *γCOP* infected and uninfected homozygous mutants, as well as infected heterozygous mutants exhibited an increased expression of the *PGRP-LC*. While this upregulation was expected for *P. aeruginosa* infected flies, the expression profile change in the uninfected homozygous males suggests that the *γCOP* mutant background induces a dysregulation of the humoral immune response in *Drosophila*. The same upregulation profile was recorded for *PGRP-LB*, an amidase that specifically degrades the Gram-negative PGN. This process downregulates the Imd pathway and provides a fine negative feedback regulation of the immune response to infection, including the modulation of the immune reactivity to ingested bacteria in the gut of the fruit flies [70]. The upregulation of this amidase in uninfected *γCOP* homozygous flies also supports the hypothesis of an immunodeficiency condition in these mutants. However, an alternative explanation could be that the fruit flies distinguish the low virulence of the infecting strain and thus an exaggerated immune response is prevented. This hypothesis is sustained by a significant upregulation of *PGRP-LB* in infected versus uninfected homozygous *γCOP* mutants.

*Fadd* and *imd* expression was inhibited in both infected and uninfected flies with different *γCOP* mutant genotypes, suggesting a disturbance of the innate immunity response in these mutants. The association of Fadd with imd is required for further activation of the Imd signaling pathway and previous studies have shown that loss of Fadd function was associated with high susceptibility of flies to Gram-negative bacteria infections [71]. Our results show that *Fadd* is strongly downregulated in all of the experimental conditions, and this could severely impair the proper activation of the Imd pathway in *γCOP* mutants. Moreover, *P. aeruginosa* infection upregulates the expression of *pirk*, a negative regulator of the Imd signaling pathways, in both mutant genotypes, thus enhancing the immunodeficiency condition in these flies. Previous studies have also shown that *pirk* overexpressing fruit flies were indeed more susceptible to Gram-negative bacterial infection than WT ones [66]. Another gene that was downregulated in infected homozygous males is *POSH*, which is essential for the properly timed activation and termination of Imd-mediated immune response [72]. These results are supporting our hypothesis that *γCOP* mutation induces a dysregulation of the innate immune response against Gram-negative bacterial infection [72].

**Figure 5:**
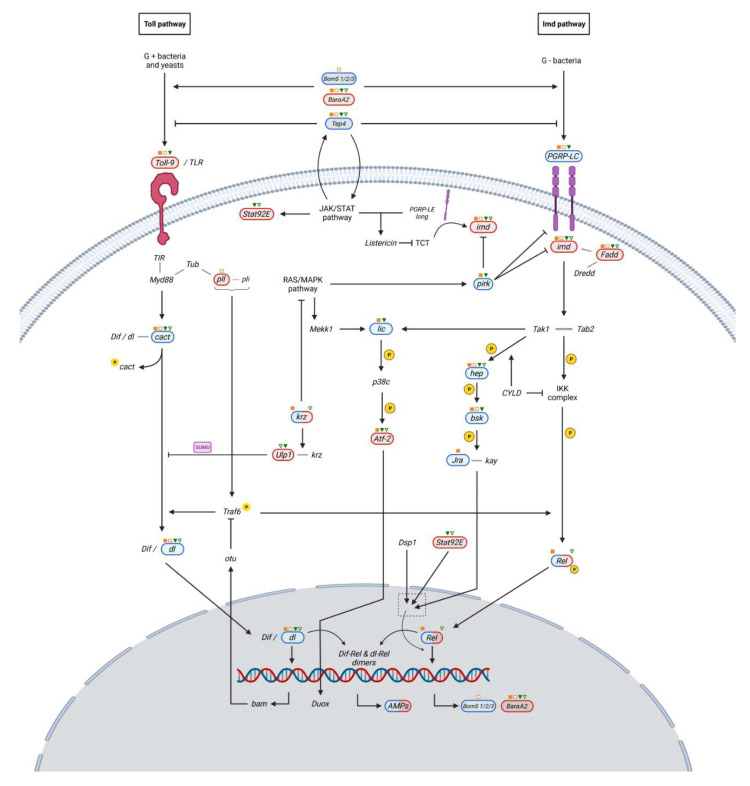
Biochemical model of interactions of Toll and Imd pathways in *D. melanogaster* as a response to microbial infection. The immune response in *D. melanogaster* involves the activation or inhibition of multiple pathways, such as RAS/MAPK, JNK, or JAK/STAT. The observed expression change corresponding to the experiment conditions and mutants is shown in blue (upregulation) or red (downregulation). With orange-filled or orange-bordered squares, we indicate that the respective gene significantly varies its expression in infected and uninfected *γCOP^14a^/γCOP^14a^* males. With green-filled or green-bordered triangles we indicate a gene that significantly varies its expression in infected and, respectively, uninfected *γCOP^S057302^*/TM6B males. Unmarked components of the pathway did not show any statistically significant expression change. Created with BioRender.com (accessed on 24 February 2022).

The main target of the Imd pathway is the transcription factor Rel, which is activated within minutes after infection, triggering the expression of AMPs genes within hours. In order to prevent an exaggerated immune response to harmless commensal microbes, the production of AMPs is tissue specific [73]. In our study, the expression of *Rel* was significantly upregulated in the homozygous infected males and downregulated in uninfected heterozygous flies. This different expression pattern is visible when all the experimental conditions are considered (FC values of infected *γCOP^S057302^*/TM6B, uninfected *γCOP^S057302^*/TM6B, infected *γCOP^14a^*/*γCOP^14a^*, uninfected *γCOP^14a^*/*γCOP^14a^*, and infected Oregon are −0.2116, −0.446, 0.439, 0.033, and, respectively, 0.0322). These data could suggest: (i) that the lack of functional *γCOP* impairs the regulation of *D. melanogaster* response intensity to a non-virulent *P. aeruginosa* strain or/and (ii) particular interaction between the Toll and Imd pathways could synergistically act to overcome some defects affecting one of the two pathways (Figure 5). It was previously shown that Toll activation may increase the level of Rel, and the *Rel* and *spz* double mutant exhibits a higher susceptibility to *E. coli* infection as compared to the *Rel* mutant fruit flies. Alternatively, the Imd pathway could regulate the Toll signaling cascade by controlling the PGRP-SA [74]. These observations are also supported by the upregulation of *Rel* and of *PGRP-SA* in the infected homozygous *γCOP* mutants.

The AMPs expression profile was predominantly downregulated in different mutant groups, suggesting that an immunodeficiency condition affecting the innate humoral immune response is mediated by AMPs in *γCOP* mutants.

However, some exceptions were noticed, i.e., *CecA2* upregulation in uninfected homozygous *γCOP* mutants, *CecB* upregulation in uninfected homozygous *γCOP* mutants and infected heterozygous *γCOP* mutants, and *Cec2* upregulation in infected/uninfected homozygous and uninfected heterozygous *γCOP* mutants. In particular, the upregulation of these cecropins in the uninfected flies suggests a defect in strict regulation of the innate humoral response in the mutant flies.

### 3.4. Toll and Imd Pathways Interplay

The increased expression levels of the cecropins in the infected heterozygous flies with a normal *Rel* level, may be explained by the possible involvement of other signaling pathways that could modulate the AMP profile, including the Toll cascade [75]. It was previously shown that *Dif* and, to a lesser extent, *dl* gene products trans-activate the *CecA1* gene in co-transfection assays [76]. In our study, *dl* was upregulated in all experimental variants and *Dif* exhibited expression profiles similar to the uninfected wild-type control (Figure 5). The upregulated or normal levels of these regulators could, at least partially, explain the increased expression levels of the three cecropins. Within the Imd cascade, the Tak1, Tab2 complex creates a bifurcation to the JNK pathway, and the complex activation is inhibited by the POSH scaffold protein [72]. This JNK bifurcation at the Tak1, Tab2 complex positively regulates the activation of the AP-1 transcription factors complex (Jra and kay) via a series of phosphorylations. The AP-1 complex translocates to the nucleus where, together with JAK/STAT controlled Stat92E and dl switch protein 1 (Dsp1), it regulates Rel-dependent expression of AMPs [77]. It was noted that simultaneous activation of Toll and Imd pathways can lead to nuclear heterodimers between Dif, dl and Rel transcription factors [78], which control the expression of AMPs associated with both pathways.

However, since a cumulative or even synergistic effect of different AMPs is needed to eliminate Gram-negative bacteria, the general AMP downregulation landscape revealed by our study indicates a defective innate humoral immune response in the *γCOP* mutants.

Considering the changes observed for the expression of genes pertaining to Toll and Imd pathways, the *γCOP* mutant background might alternatively modulate other members of the complex innate immunity network, to allow the fruit flies to resist microbial aggression. In the extracellular space, proteins regulated by the Toll pathway, such as BomS1, BomS2, BomS3 [79], or Baramicin proteins (BaraA1, BaraA2) [80], and by the JAK/STAT pathway, involved in regulation of Tep4 via a positive feedback loop [64,81], act as immune response regulators upstream of transmembrane receptors. In our study, an increased expression of *BomS1*, *BomS2* and *BomS3* was observed in uninfected homozygous mutants. This family of secreted peptides is known to mediate the innate immune response activated by *Drosophila* Toll receptors upon pathogen recognition and it is involved in fruit fly’s resistance to infection [79,82].

All infected and uninfected fruit flies exhibit upregulated levels of *Tep4*, whose product is involved in the opsonization and elimination of pathogens and in the amplification of the inflammatory response in vertebrates. Tep4 also plays a role in regulating apoptosis, metabolic activities, and pathophysiological effects in fruit flies challenged by *Photorhabdus* infection [83,84]. The upregulation of *Tep4* in both infected and uninfected fruit flies could suggest that the *γCOP* mutant phenotype triggers augmented and potentially deleterious inflammatory responses to less virulent and commensal microbes. Both infected and uninfected heterozygous *γCOP* mutants exhibit a downregulation of *Stat92E* from the JAK−STAT signaling pathway, which was previously shown to be involved in *D. melanogaster* response against DNA viruses [85]. *Stat92E* downregulation could partially explain the downregulation of *Rel* observed in uninfected heterozygous males.

As already shown in Figure 3 and Figure 4, *Traf6* is regulated by both Toll and Imd pathways, facilitating nuclear translocation of each pathway’s final transcription factors [50,67]. Both the krz-Ulp1 sumoylation complex [52], which inhibits nuclear translocation of Dif and dl, and pirk, which inhibits imd activation by the transmembrane receptors [66], are linked to the RAS/MAPK pathway. The RAS/MAPK pathway is involved in activation of the stress response regulating protein Mekk1, and along with the Tak1 and Tab2 complex, activates the serine/threonine kinase licorne (lic), leading to p38c and Atf-2 phosphorylation and resulting in *dual oxidase (Duox)* expression [86]. The p38 mitogen-activated protein (MAP) kinase signaling cascade is known to be involved in the physical, chemical, and biological stress response and immunity in different species. Among other substrates, the p38 kinases phosphorylate the ATF family transcription factors. In our study, *Atf-2* was downregulated in the infected experimental groups, as well as in the uninfected heterozygous males. The decreased expression levels of *Atf-2* could be linked to a lower production of ROS, as a result of a decreased level of the ROS-producing enzyme Duox [86] (*Duox* exhibited a FC of −0.145 in infected *γCOP^14a^*/*γCOP^14a^*, almost reaching the significance threshold with a *p* value of 0.088).

## 4. Materials and Methods

### 4.1. D. melanogaster Stocks

To perform the experimental infection and subsequent microarray experiment we employed young adult males from three strains of *D. melanogaster* that were maintained on the Nutri-Fly Bloomington Formulation medium (Genesee Scientific, San Diego, CA, USA). These strains are: 1. Oregon, standing for the standard WT strain; 2. *γCOP^S057302^*/TM6B harboring the insertion of *P{lacW}γCOP^S057302^* transposon in the 5′UTR region of *γCOP* gene (GenBank accession number AJ492220) and causing embryo lethality; 3. The homozygous *γCOP^14a^*/*γCOP^14a^* males were harvested from *γCOP^14a^*/TM6B strain and contain the hypomorphic *γCOP^14a^* allele (GenBank accession number DQ279402). Both mutant lines have *white* defective genetic background.

### 4.2. Bacterial Strain

The *P. aeruginosa* ATCC 27853 strain was cultivated on Cetrimide agar at 37 °C and following 24 h of cultivation, bacterial suspensions of 9 × 10^9^ CFU/mL were obtained.

### 4.3. Bacterial Ingestion Assay

WT Oregon, *γCOP^S057302^*/TM6B, and *γCOP^14a^*/*γCOP^14a^* males were starved for several hours prior to being placed in vials containing 5% sucrose agar covered with sterile paper blotted with either 230 µL of 5% sucrose solution containing 9 × 10^9^ CFU of *P. aeruginosa* suspension (infected replicates) or 230 μL of 5% sucrose (uninfected replicates).

A total of 20 Oregon uninfected replicates were considered as reference; for each of the *γCOP^S057302^*/TM6B and *γCOP^14a^*/*γCOP^14a^* infected and uninfected experimental variants four biological replicates were used, as well as for the infected Oregon males. Every biological replicate comprised of 25 to 27 individuals.

The males were allowed to feed for about 63 h at 25 °C, then, a total of 25 males/replicate were placed in 1.5 mL Eppendorf tubes, covered with 300 mL TRIzol, and shipped to FlyChip Facility (Cambridge Systems Biology Centre, Cambridge, UK).

### 4.4. Microarray Project

The *D. melanogaster* samples (40 biological replicates) were processed at FlyChip according to their standard protocol. Briefly, medium-scale RNA extraction was performed in the first step; then, the 20 control Oregon RNA samples were pooled together and converted into double-stranded DNA used as a reference to compare all experimental conditions against the control. All the samples were submitted to Klenow labelling of 1 µg double-stranded DNA derived from total RNA and subsequent hybridization to amino-modified long oligonucleotide microarrays, using the FL003 (FlyChip_long_oligonucleotide_003—INDAC) array format. The fluorescent spots were analyzed with a GenePix Axon scanner (5 μM pixel resolution).

### 4.5. Gene Expression Data Set

Microarray data are available online at NCBI’s Gene Expression Omnibus (GEO), accession number GSE80084. Normalization and quality control steps were performed using *vsn* [87] and *limma* [88] software packages (Bioconductor). The resulting values are expressed as log_2_ ratios of sample/control (log_2_FC values).

### 4.6. Microarray Data Analysis

Heatmaps summarizing the up− and downregulated genes associated with Toll and Imd pathways, as well as the overview of the differential expression of immune genes in *γCOP* mutants, were generated with GraphPad Prism 5.03 software (GraphPad Software, La Jolla, CA, USA) using the log_2_FC values.

## 5. Conclusions

This insight into the whole-body transcriptome of *D. melanogaster* males with specific *γCOP* mutant backgrounds revealed that the expression of at least 52 genes of the Imd and Toll pathways and their interactors were significantly modulated upon infection with *P. aeruginosa*. Remarkably, in both infected and uninfected mutant males, the expression pattern of a vast majority of the genes from both pathways is affected when compared to the uninfected Oregon control. A very interesting aspect is that none of these genes are significantly modulated in infected Oregon males. This led us to hypothesize that natural microbiota might act as an environmental trigger in *γCOP* mutant background, a condition that signals an alert state. The peculiar microarray pattern may suggest that genes from Toll and Imd pathways respond to this alert by adjusting their transcription rate even in the absence of experimental infection with *P. aeruginosa*. One scenario is that, in the absence of enough functional *γCOP* product, the host increases the stringency of the feedback control of commensal microbiota, by triggering an erratic immune response.

Further studies are needed to confirm and refine the contribution of both host’s genetic background and different virulence factors to bacterial pathogenicity and host immune response. In our experimental design, we have selected a laboratory *P. aeruginosa* bacterial strain with low virulence administered by ingestion, in order to evaluate the ability of adult flies to adequately sense and respond to the presence of Gram-negative ubiquitous bacteria relative to their *γCOP* genotype. In natural conditions, fruit flies seem to be able to detect Gram-negative components, such as LPS, which is sensed through the transient receptor potential (TRP) channels (e.g., the TRPA1 protein), and thus avoid eating food contaminated with LPS and bacteria [89]. However, whether the activated receptors further activate the fruit fly’s immune response to a potential infection remains to be elucidated. Studies performed on mice have shown that the deletion of TRPA1 decreases pain and inflammation induced by LPS, suggesting that detection of bacterial components in food modulates the innate response to Gram-negative pathogens [90]. Complementarily, research using highly virulent *P. aeruginosa* strains administered by direct injection in the hemolymph [91] could help to elucidate the role of *γCOP* mutations in the innate immune response to Gram-negative infections. The *γCOP* mutations are affecting the fruit fly’s ability to recognize the pathogen-associated molecular patterns and to further activate the signal transduction cascades and the transcription of effector genes in the Toll cascade. Both inhibitory (downregulation of *GNBP1*, inhibition of *SPE* and *Toll-9*, upregulation of *cact*, *wntD*, and *Gprk2*) and activating (inhibition of *Ulp1*/*krz*, upregulation of *PGRP*-*SA*, *PGRP*-*SD,* and *ModSP*) effects were noticed, suggesting a dysregulation of this important innate immunity pathway. The same dual effects were recorded for the Imd cascade, whose expression profiles suggest either inhibition (upregulation of *PGRP-LB*, inhibition of *imd* and *Fadd*, upregulation of *pirk*) or activation (increased expression of the *PGRP*-*LC* and downregulation of *POSH*). The variable expression profile of transcriptional factors and AMPs in different experimental groups demonstrates the complex interactions between Toll and Imd, as well as with other signaling pathways.

Interestingly, *γCOP^14a^/γCOP^14a^* males are also unable to compensate for the conditional sterility of *γCOP^14a^/γCOP^14a^* females in the respective crosses [30]. We argue that *γCOP* is the most probable genetic determinant herein, since comparative data we obtained for viability, fertility, and infection tests points to a similar behavior of the inquired *γCOP* alleles. Basically, *γCOP^14a^* behaves as a hypomorph, and therefore is a less severe allele than the *γCOP^S057302^* null, but heterozygous *γCOP^S057302^*/TM6B exhibit less severe phenotypes of sterility and immune sensitivity compared to *γCOP^14a^/γCOP^14a^* males.

Overall, our data suggest that the *γCOP* background interferes with the Imd and Toll signaling pathways at different levels. The *γCOP* mutations disturb the fine regulation of the innate immune response and hence the fruit fly’s capability to adequately respond to Gram-negative bacterial infections.

Our results reveal *in premiere* that mutations of the *γCOP* gene impair not only the adaptative immune response, as in humans and mice, but also the innate immune response of *D. melanogaster*, reinforcing the power of this organism for modeling infections.

Purportedly, the prognostic of some bacterial infections should be estimated not only by the classical clinical procedures, but also by a quick screening of patients for *γCOP* mutations, as inferred from recent medical research data [19].

## Figures and Tables

**Table 1:** The genes associated with Toll, Imd, and Imd-JNK pathways with significant modulation of expression in at least one experimental condition.

Toll Pathway		
Gene Name	Gene Symbol	Biological Process
*Baramicin A2*	*BaraA2*	defense response
*Bomanin Short 1*	*BomS1*	response to bacteria
*Bomanin Short 2*	*BomS2*	defense response
*Bomanin Short 3*	*BomS3*	response to bacteria
*cactus*	*cact*	negative regulation of Toll signaling pathway
*Defensin*	*Def*	humoral immune response
*dorsal*	*dl*	dorsal/ventral axis specification
*Drosomycin*	*Drs*	defense response to protozoa
*Drosomycin-like 1*	*Drsl1*	defense response to fungi
*Gram-negative bacteria binding protein 1*	*GNBP1*	carbohydrate metabolic process
*G protein-coupled receptor kinase 2*	*Gprk2*	protein phosphorylation
*Gram-positive Specific Serine protease*	*grass*	proteolysis
*kurtz*	*krz*	locomotory exploration behavior
*modular serine protease*	*modSP*	proteolysis
*Metchnikowin*	*Mtk*	defense response to Gram-positive bacteria
*Peptidoglycan recognition protein SA*	*PGRP-SA*	innate immune response
*Peptidoglycan recognition protein SD*	*PGRP-SD*	innate immune response
*pelle*	*pll*	Toll signaling pathway
*Spatzle-Processing Enzyme*	*SPE*	defense response to Gram-positive bacteria
*spheroide*	*sphe*	proteolysis
*spatzle*	*spz*	defense response to Gram-positive bacteria
*Thioester-containing protein 4*	*Tep4*	innate immune response
*Toll-9*	*Toll-9*	signal transduction
*Ulp1*	*Ulp1*	negative regulation of Toll signaling pathway
*wnt inhibitor of Dorsal*	*wntD*	defense response to Gram-positive bacteria
**Imd Pathway**		
**Gene Name**	**Gene Symbol**	**Biological Process**
*Attacin-A*	*AttA*	humoral immune response
*Attacin-B*	*AttB*	humoral immune response
*Attacin-C*	*AttC*	antibacterial humoral response
*Attacin-D*	*AttD*	response to wounding
*bendless*	*ben*	positive regulation of tumor necrosis factor-mediated signaling pathway
*Cecropin pseudogene 1*	*Cec-Ψ1*	pseudogene
*Cecropin 2*	*Cec2*	pseudogene
*Cecropin A2*	*CecA2*	antibacterial humoral response
*Cecropin B*	*CecB*	defense response to Gram-positive bacteria
*Diptericin A*	*DptA*	response to bacteria
*Diptericin B*	*DptB*	response to wounding
*effete*	*eff*	germ-line stem cell population maintenance
*Fas-associated death domain*	*Fadd*	peptidoglycan recognition protein signaling pathway
*Immune deficiency*	*imd*	response to bacteria
*Peptidoglycan recognition protein LB*	*PGRP-LB*	innate immune response
*Peptidoglycan recognition protein LC*	*PGRP-LC*	regulation of synaptic plasticity
*poor Imd response upon knock-in*	*pirk*	negative regulation of peptidoglycan recognition protein signaling pathway
*Plenty of SH3s*	*POSH*	response to peptidoglycan
*Relish*	*Rel*	peripheral nervous system neuron development
**Imd-JNK Pathway**		
**Gene Name**	**Gene Symbol**	**Biological Process**
*Activating transcription factor-2*	*Atf-2*	positive regulation of transcription by RNA polymerase II
*Octopamine receptor in mushroom bodies*	*Oamb*	cellular calcium ion homeostasis
*basket*	*bsk*	cellular response to oxidative stress
*hemipterous*	*hep*	positive regulation of cell death
*Jun-related antigen*	*Jra*	wound healing
*licorne*	*lic*	JNK cascade
*Phospholipase C at 21C*	*Plc21C*	lipid metabolic process
*Signal-transducer and activator of transcription protein at 92E*	*Stat92E*	receptor signaling pathway via JAK-STAT

## Data Availability

Data regarding *γCOP^S057302^* and *γCOP^14a^* alleles are available in GenBank/NCBI, accession number AJ492220 and, respectively, accession number DQ279402. Microarray data are available online at NCBI’s Gene Expression Omnibus (GEO), accession number GSE80084.
